# Supplementary material for: Forecasting Seizure Likelihood With Wearable Technology
Source: Front Neurol. 2021 Jul 15;12:704060. doi: 10.3389/fneur.2021.704060 (PMC8320020; doi:10.3389/fneur.2021.704060)
Supplement: Supplementary file 1 [file Data_Sheet_1.docx]

**Supplementary material**


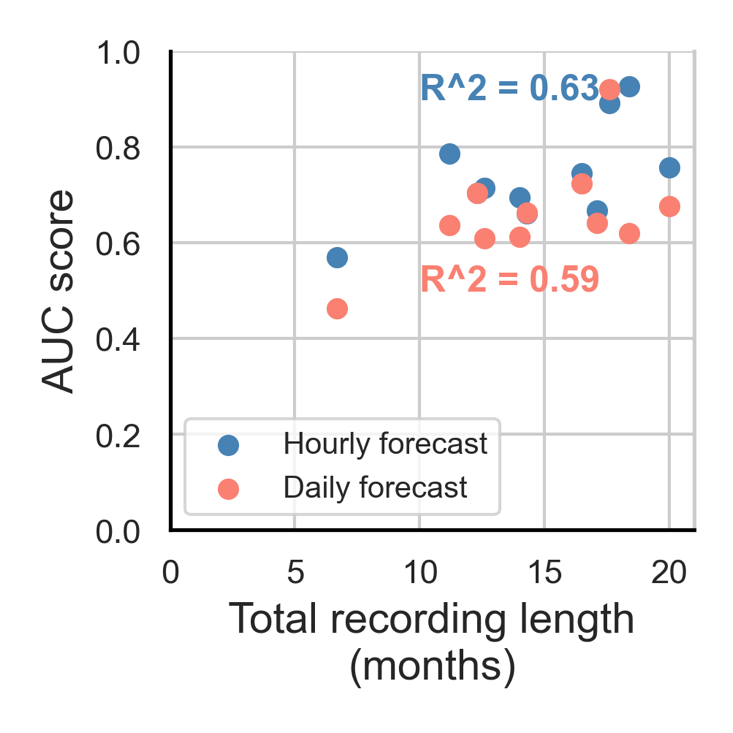


**Supplementary Figure 1.** The relationship between AUC scores (shown in Figure 2) and recording length (months), shown for all participants. AUC scores were shown for both the hourly forecaster (blue) and the daily forecaster (orange). The pearson correlation coefficient (R^2^) of each forecaster is also shown.


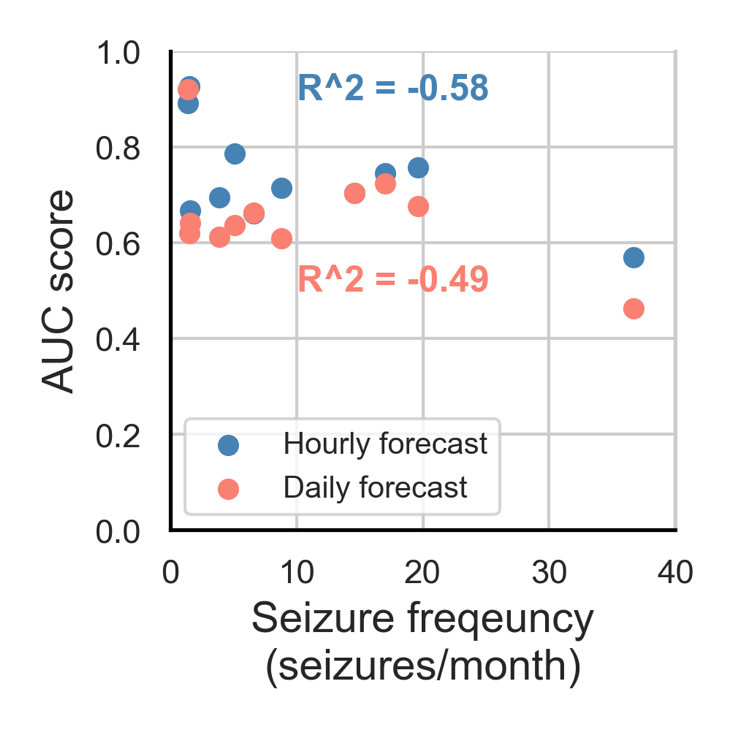


**Supplementary Figure 2.** The relationship between AUC scores (shown in Figure 2) and seizure frequency (seizures/months), shown for all participants. AUC scores were shown for both the hourly forecaster (blue) and the daily forecaster (orange). The pearson correlation coefficient (R^2^) of each forecaster is also shown.
